# Policy Makers’ Perceptions on Implementation of National Action Plans on Antimicrobial Resistance in South Africa and Eswatini Using Coordination, Accountability, Resourcing, Regulation and Ownership Framework (2018–2019)

**DOI:** 10.3390/antibiotics14070696

**Published:** 2025-07-11

**Authors:** Kholiwe Shabangu, Sabiha Yusuf Essack, Sinegugu Evidence Duma

**Affiliations:** 1 School of Pharmaceutical Sciences, College of Health Sciences, University of KwaZulu-Natal, Durban 4000, South Africa; 2 Antibiotic Research Unit, School of Pharmaceutical Sciences, College of Health Sciences, University of KwaZulu-Natal, Durban 4000, South Africa; essacks@ukzn.ac.za; 3 School of Nursing and Public Health, College of Health Sciences, University of KwaZulu-Natal, Durban 4000, South Africa; dumas1@ukzn.ac.za

**Keywords:** antimicrobial resistance, national action plan, One Health approach, CARRO framework

## Abstract

**Background**: Antimicrobial resistance (AMR) is a global threat that affects humans, animals, plants, the environment, societies, and economies—requiring urgent coordinated action. In May 2015, the World Health Assembly (WHA) adopted the Global Action Plan (GAP) on AMR, urging member states to develop and implement their own National Action Plans (NAPs) using a One Health approach. **Objective**: Both South Africa and Eswatini have developed NAPs and are currently in the implementation phase. However, no study has explored policymakers’ perceptions regarding NAP implementation particularly concerning coordination, accountability, resourcing, regulation and ownership. **Methods**: This qualitative study employed a narrative approach to explore these perceptions in South Africa and Eswatini. A total of 36 key informants were recruited using purposive and snowball sampling methods. Data was collected between November 2018 and March 2019 and transcribed verbatim. **Results**: Findings revealed that while governance structures for implementing NAPs exist in South Africa and Eswatini, several critical areas require urgent attention. These include limited accountability across One Health sectors, the absence of dedicated national budgets for NAP implementation, poor intra- and inter- ministerial coordination, weak medicine regulation and enforcement, and delayed multisectoral ownership of the NAPs. **Conclusions**: To address AMR effectively, both countries must allocate dedicated budgets, improve multisectoral integration, and strengthen regulatory frameworks regarding antimicrobial access and use across all One Health sectors. A firm commitment from all sectors is required—not just formal endorsement of the NAPs—to ensure sustainable implementation and ownership.

## 1. Introduction

Antimicrobial resistance (AMR) is a major global threat to humans, animals, plants and the environment and it undermines the safety of food systems and ecosystems [1]. In 2019 alone, an estimated 495 million deaths were associated with bacterial AMR infections [2]. If left unaddressed, AMR could push up to 24 million people into extreme poverty by 2030 [3]. While the rate of AMR continues to increase alarmingly, the development of new antibiotics has slowed considerably [4]. The current supply of antimicrobials is inadequate to match the pace of resistance, as older antibiotics lose their effectiveness [5]. Therefore, AMR requires a robust, and collaborative global response [6]. Significant progress in fighting AMR has largely occurred in high income countries (HICs) through comprehensive health sector strategies, while low-middle-income countries (LMICs) continue to lag behind [6].

In recognition of this global challenge, the World Health Assembly (WHA) adopted the Global Action Plan (GAP) on AMR in 2015. As part of this resolution, all World Health Organization (WHO) member states committed to developing and implementing their own National Action Plans (NAPs) based on the One Health approach by 2017 [7,8,9,10].

To support these efforts, WHO developed a country self-assessment tool to help governments monitor and report progress in developing and implementing their AMR NAPs [9]. Although this tool plays an important role in maintaining momentum and ensuring global policy alignment, it has faced criticism for its subjective nature and the lack of third-party triangulation and validation of the scores [11]. As of 2024, all 47 African WHO member states had developed AMR NAPs. However, the implementation of these plans remains largely aspirational. Many are not integrated into national health strategies or fully funded, with only 20% of AMR NAPs globally receiving full funding or having the appropriate systems in place to be effectively executed [7,12,13,14,15]. Studies performed in Brazil, Ghana, Philippines, South Africa and Eswatini report insufficient government commitment and chronic underfunding, with implementation efforts often reliant on donor support [4,13,16,17].

No single country or sector can solve the AMR challenge alone. A comprehensive, large-scale One Health approach is needed across human and animal health, food production, environmental management, water and sanitation, education, and trade sectors at international, regional, and national levels [5]. The One Health approach is a collaborative, multisectoral and transdisciplinary approach that seeks to sustainably balance and optimize the health of people, animals, and the environment [18]. Unfortunately, AMR competes for resources with other high-priority health concerns such as HIV, tuberculosis, malaria, and universal health coverage (UHC) [7].

South Africa and Eswatini are neighboring countries in sub-Saharan Africa and both are WHO member states. However, they differ in economic status: South Africa is an upper-middle-income country (UMIC) with a population of approximately 62 million while Eswatini is a low-middle-income country (LMIC) with a population of about 1.2 million [19,20]. South Africa first developed its NAP on AMR—the National AMR Strategic Framework for 2014-2024—in 2014, which was revised in 2018 to include the animal health sector [21,22]. Eswatini published its National Antimicrobial Resistance Containment Strategic Plan 2018—2022 in 2020, with implementation planned through 2025 [23].

South Africa began participating in WHO’s country self-assessments since 2018, while Eswatini joined in 2020 [24,25]. Reports from both countries show varied implementation levels across One Health sectors and limited integration of activities. However, no prior research has explored policymakers’ perceptions of NAPs on AMR implementation using the Coordination, Accountability, Resourcing, Regulation and Ownership (CARRO) framework [24,25]. This study aimed to explore policymakers’ perceptions of NAPs on AMR implementation in South Africa and Eswatini, guided by the CARRO framework and employing a One Health lens. (See Anexure 1 for interview guide attached at the end of the manuscript).

The CARRO tool (interview guide) was developed by a team of AMR experts from Sweden and South Africa to assess the implementation process of NAPs on AMR. It was validated through a consultative process with researchers from pharmaceutical sciences, veterinary medicine, medical laboratory specialists, infection prevention specialists, infectious disease specialists, water affairs, and environment health. The tool assessed the following domains:

1. Whether a national coordinating team was in place and represented all One Health sectors.
2. Accountability of each sector to NAP activities.
3. Availability of financial and human resources.
4. Regulation of antimicrobial access and use across One Health sectors.
5. Ownership of NAPs, demonstrated through formal endorsement and active implementation.

This study provides insights into the perspectives of policymakers in South Africa and Eswatini regarding the effectiveness, gaps, and challenges associated with NAPs on AMR implementation, using the CARRO framework as a guiding tool.

## 2. Methods

A qualitative research design using a narrative approach was employed to explore policymakers’ perspectives on progress made in implementing NAPs on AMR using the One Health approach in South Africa and Eswatini. This design was appropriate for providing in-depth insights into the perspectives of those directly involved in AMR policy and implementation.

### 2.1. Research Setting

The study was conducted across three provinces in South Africa, namely KwaZulu—Natal, Gauteng, and Western Cape—and in two regions in Eswatini: Manzini and Hhohho. These areas were selected based on the geographical locations of the key informants (KIs) targeted for interviews.

Table 1 presents a summary of the study participants’ profiles across different One Health sectors. Notably, there were fewer participants from the environmental sector compared to the health and agriculture sectors.

### 2.2. Sampling, Participant Recruitment, and Selection

A total of 36 key informants were interviewed. Initially, 26 were purposefully selected (14 from South Africa and 12 from Eswatini) based on their expertise in AMR and involvement in the development or implementation of NAPs.

KIs were drawn from national AMR governance structures, including the Ministerial Advisory Committee (MAC) under the South African National Department of Health (NDoH) and the National Antimicrobial Resistance Containment Committee (NAMRCC) under the Eswatini Ministry of Health (MoH).

Snowball sampling was also used, with each KI asked to recommend additional informants. Ten more participants (six from South Africa and four from Eswatini) were recruited this way. Potential participants were contacted via e-mail informing them about the study and requesting their participation. Those who agreed were then requested to provide a date and time convenient to them to be interviewed and researchers traveled to their offices. All interviews were conducted with informed consent, audio recorded with permission, and supplemented with field notes to capture non-verbal cues. Participants were also informed of their right to withdraw from the interviews at any time. All interviewees gave written consent prior to participation in the study. Confidentiality was ensured using coded identifiers in accordance with the Helsinki Declaration [26].

Data collection continued until data saturation was achieved, meaning no new themes emerging from the interviews. This final sample size *(n* = 36) was considered adequate because the intention was not to quantify the results but to draw the richness of the in-depth information about the participants’ perceptions on progress of implementing NAPs on AMR in a One Health approach using the CARRO framework.

### 2.3. Pilot Study

A pilot study was conducted with two participants from South Africa’s health sector to assess the clarity and relevance of the interview questions. The pilot helped identify any potential ethical concerns and allowed refinement of the guide before full-scale data collection.

Preliminary analysis of the pilot data confirmed the questions yielded meaningful responses. Data collected from the pilot study was included in the main study for data analysis as there was no possibility of data contamination. This is common in qualitative studies where there is no fear of data contamination [27].

### 2.4. Data Collection

Data was collected from November 2018 to January 2019 in South Africa, and from February to March 2019 in Eswatini. Face-to-face interviews were conducted using a semi-structured interview guide based on the CARRO framework and aligned with the study objectives. All interviews were conducted in English, in the informants’ workplaces. Each session lasted between 60 and 90 min and was conducted by two researchers, one of whom had expertise in qualitative research expert. KIs were asked open-ended questions about their views on the progress of implementation of NAPs on AMR using a One Health approach in the context of the CARRO framework with follow-up questions used to elicit more detailed information or clarifications on specific points raised. The interview guide included questions covering the five CARRO framework domains: coordination, accountability, resourcing, regulation, and ownership.

### 2.5. Data Management

Data collection, transcription and analysis were conducted concurrently, as recommended by Creswell 2013 [28]. The interviews were transcribed verbatim by Shabangu K. within 24 to 48 h of data collection into a word document. A member of the research team reviewed each transcript against the audio recordings to ensure accuracy.

All identifying information was removed during transcription to maintain confidentiality. Each transcribed interview was coded according to participant number, One Health sector and country. This coding system allowed for easy identification while preserving anonymity.

Transcripts were uploaded into QDA international NVivo version 12 software for storage and management of data for analysis. Data were stored on a password-protected computer, with regular backups made to a secure, encrypted external hard drive. Access to the data was limited to the research members who were directly involved in the analysis.

### 2.6. Data Analysis, Scientific Rigor, and Trustworthiness

Thematic data analysis was conducted following Braun and Clarke’s methodology [29]. Researchers familiarized themselves with the data through repeated readings. Line by line analysis of the data was performed to identify emerging codes, which were used to create labels and nodes within NVivo. To ensure reliability, intercoder agreement process was then employed between the Shabangu K. and Sabiha S. using four transcripts to test if analysis of the transcripts yielded similar codes. This process is undertaken for reliability of the data, and 87.4% agreement was achieved which is acceptable according to Miles and Huberman 1994 who recommend a 70% agreement [27]. Developed codes were shared with Duma S., a qualitative research expert, who conducted a rigorous comparative analysis of the data to verify the identified codes against raw data to ensure credibility of the study [30]. To confirm accuracy, the analyzed data was shared with study KIs in South Africa and Eswatini through a group presentation to confirm if it were a true reflection of their perceptions as recommended by Creswell 2013 [28]. The codes were grouped according to similarities within and across cases, resulting in the development of five themes, which were then supported by quotes from transcribed data. These themes represent the perceptions of policymakers on the progress of implementation of NAPs on AMR using the CARRO measure.

## 3. Findings

Five main themes and related subthemes emerged from data analysis as follows: 1. Multi-disciplinary governance structures for implementing NAPs on AMR in place. 2. Limited accountability of One Health sectors. 3. Inadequate resources for effective implementation. 4. Non-compliance with medicines regulations. 5. Lack of multisectoral ownership.

### 3.1. Multidisciplinary Governance Structures for Implementing NAPs on AMR in Place

Both countries had established governance structures to coordinate NAPs on AMR implementation. In South Africa, the Ministerial Advisory Committee (MAC) leads this process, while in Eswatini, the National Antimicrobial Resistance Containment Committee (NAMRCC) assumes this role as illustrated below:

“There is a coordinating committee in place and it comprises of people from health, agriculture and natural resources and we also bring in the environment, WHO for technical guidance and there is FAO and OIE representatives”.

-P1, Health, Eswatini

“The MAC for AMR which has members from health, agriculture, veterinary sector, and the environment sector provides leadership and guidance to bring everyone and everything together and to prioritize which activities needs to be implemented first on the NAP”.

-P2, Health, South Africa

### 3.2. Limited Accountability of One Health Sectors

KIs revealed a lack of accountability within and across sectors, particularly regarding awareness and implementation at operational levels as illustrated in the following extracts:

“We have not been effective in our awareness education programs. The government has not supported AMR awareness education programs as part of the activities to reduce AMR through awareness of overuse and misuse of antimicrobials in humans and animal health”.

-P5, Health, South Africa

“We have a coordinating team, but it has limitations in terms of practical implementation; because remember these things are happening at the hospitals or at veterinary clinics, or at the farmer level and none of these people are implementing the strategy let alone know about it, therefore there is no one accountable”.

-P10, Agriculture, Eswatini

### 3.3. Inadequate Resources for Effective Implementation

Two subthemes emerged under this category: the absence of dedicated budgets and limited human resources. This is illustrated below:

#### 3.3.1. Lack of Dedicated Budget for the Implementation of NAP Activities

Findings revealed that there was no dedicated budget from governments to implement NAPs on AMR and both countries relied on donor funding for the implementation of activities, as illustrated in the following extracts:

“There is no budget that is called AMR budget. There is no portfolio that is called AMR. Every year we make requests for new areas that we want to fund but unfortunately, we have not been successful.”

-P12, Agriculture, South Africa

“Absolutely zero, and I can say that because I have been personally trying for the last number of years to convince the Department of Agriculture, Land Reform and Rural Development (DALRRD) to provide funding for a national surveillance program in the animal health sector, and it still does not exist.”

-P10, Agriculture, South Africa

“We do not have earmarked resources for AMR. If you go into our budget, there is no line that says AMR. AMR is not funded at all; it is funded by default.”

-P1, Health, Eswatini

The following quotes illustrate reliance on donor funding for the implementation of NAP activities like AMR surveillance in both countries.

“There was a very good antibiotic resistance surveillance program in 2007 in animal health which was funded by joint Swedish and South African grant and when it stopped, surveillance fell by the side.”

-P10, Agriculture, South Africa

“In terms of resources, we tend to be lacking a lot because, most of these activities implemented are partner driven, it is highly partner or donor- driven”.

-P2, Health, Eswatini

#### 3.3.2. Limited Human Resources

Participants reported workforce shortages across several critical professions.

“There is a shortage of qualified microbiologists particularly in the state sector, infection prevention physicians. I think there is a shortage of pharmacists with a clinical pharmacy qualification to manage stewardship programs.”

-P4, Health, South Africa

“We do not have microbiologists, infectious disease specialists, or even pharmacists which are important in AMR containment. The role of these professionals cannot be over-emphasized in AMR surveillance and antimicrobial stewardship implementation.”

-P8, Health, Eswatini

“The technical level is depleted; we have not employed a new state veterinarian for the past two years. We have a huge gap between a veterinarian and animals that need diagnosis and treatment.”

-P11, Agriculture, South Africa

### 3.4. Non-Compliance with Medicines Regulations

Findings revealed that both countries had medicine legislations in place to curb AMR; however, both countries reported non-compliance to the legislations in the different One Health sectors. The challenges ranged from having two opposing legislations for regulating medicines access and use in animal health in South Africa and failure to enforce medicine legislation in Eswatini as illustrated in the extracts below:

“One of the difficult things in South Africa is that we have two sets of laws that we abide by for registration and use of medicines in animal health, i.e., Act 101 of 1956 (Medicines and Related Substance) and Act 36 of 1947 (The Fertilizers, Farm Feeds, Seeds and Remedies). Act 101 of 1965 is controlled and there must be a script involved and then Act 36 of 1947 allows wholesalers to sell antibiotics to farmers on request without the proper procedures of antimicrobial sensitivity testing being followed. Anybody can go into a cooperative shop and buy nitrofurantoins, sulfonamides, oxytetracyclines without a prescription.”

-P14, Agriculture, South Africa

Despite the existence of new medicines legislation in Eswatini, weak enforcement of regulations was identified as a key challenge hindering the implementation of the NAP on AMR, as illustrated in the following excerpt:

“We have our new Medicines and Related Substances Act of 2016 in place, but we currently do not have regulations in place to enforce it. Our retail pharmacists know that antibiotics are not over-the-counter (OTC) medicines, but there is a danger of them selling them to clients as OTC medicines because there are no punitive measures in place to prevent that from happening.”

-P2, Health, Eswatini

### 3.5. Lack of Multisectoral Ownership

Respondents described delayed endorsement and weak political commitment as key barriers to ownership as illustrated in the extracts below:

“The first NAP on AMR was only signed by the Minister and Director General of Health which meant commitment was only from the health sector. It was then reviewed and now we have signed the strategy and have co-ownership on the strategy with health.”

-P11, Agriculture, South Africa

“We have had a joint external evaluation for the International Health Regulations (IHR), and we did not perform well; we were rated very low on AMR because the strategy had not been signed by one (the environment) sector but now, I am happy that it has been signed.”

-P6, Health, Eswatini

“The challenge was at the final stage in terms of immediate owning the document and this delayed commencement of the implementation process.”

-P12, Agriculture, Eswatini

“I might say lack of interest from the national government. There was a strong leadership from the office of the Director General earlier on, but it has gone to the back bench, it has really dropped.”

-P7, Health, South Africa

## 4. Discussion

This study provides insights into policymakers’ perspectives of the implementation progress of NAPs on AMR in South Africa and Eswatini, using the CARRO framework.

Both countries have established national governance structures formed in a multidisciplinary approach responsible for coordinating the implementation process of NAPs on AMR. These governance structures are both centrally coordinated by the Department of Health in both countries. The establishment of governance structures in a multidisciplinary approach is a step in the right direction for effective implementation of NAPs on AMR as recommended by WHO [10,31].

Although the governance structures were found to be in place, it was notable from the profile of the members that there was minimal representation (if any) of the environmental health sector in both countries affecting the sector’s visibility and contribution to the One Health approach as it is responsible for environmental pollution, water, and sanitation. The visibility of the environment sector is important in AMR as it advocates for use of clean and safe water in communities, hygiene, and proper sanitation to prevent infectious diseases that could trigger increased antibiotic use and subsequently AMR [32]. Similar gaps in environmental sector involvement have been reported in studies from the Philippines and Tanzania, where limited integration hindered NAP execution [4,33]. Ogyu et al. 2020 and Lota et al. 2022 also reveal that more policies are aimed at human and animal sectors with limited AMR-related policies in the environment and plant sectors [4,34]. There should be inclusion of environmental representatives in coordinating committees to ensure AMR activities are prioritized in environmental policies and programs to support the sustainability of AMR initiatives [4].

Both countries showed limited accountability for implementing NAP activities. In Eswatini, operational staff were often unaware of the NAP’s existence, while in South Africa, awareness campaigns were hindered by insufficient government support. This reflects the broader challenge of translating strategic commitments into tangible, sector-specific actions. To ensure accountability, the NAPs specify activities to be carried out by each One Health sector within set timeframes to curb AMR [21,35]. The WHO implementation handbook emphasizes that NAPs should outline clear, time-bound responsibilities for each One Health sector [31]. Since it is not feasible to implement all activities simultaneously, coordinating bodies must prioritize key interventions and monitor progress accordingly. Effective implementation of activities to mitigate AMR requires a prioritized interdisciplinary effort from all One Health stakeholders.

The absence of dedicated budgets in both countries emerged as a major barrier to implementation. Without domestic financial support, most NAP activities rely on external funding—raising sustainability concerns. These findings echo studies from the Philippines and Tanzania, where governments failed to allocate sufficient resources to implement AMR strategies [4,36]. Eswatini benefits from external donors such as the Fleming Fund [14,35,37]. Contrary to our findings, Singapore has dedicated financial resources, but the implementation of their NAP remains complicated due to challenges such as low public awareness, disagreements between human and animal sectors, and political and cultural aspects influencing AMR containment [38]. In both South Africa and Eswatini, surveillance programs have collapsed when donor funding ended. This concern is mirrored in studies from Tanzania and the Philippines, where policymakers emphasized that donor-driven implementation threatens long-term continuity [4,36,39]. Human resource shortages in both countries further hamper progress. The lack of trained microbiologists, pharmacists, infectious disease specialists, and veterinarians limits the implementation of stewardship programs and surveillance systems. Similar workforce gaps have been documented in the Philippines, Ghana, and Tanzania, suggesting a systemic challenge in LMICs [4,33,40].

Respondents reported that the two legislations responsible for regulating medicines access and use in animal health in South Africa were a challenge in curbing AMR as they were competing with each other. Act 36 of 1947 allowed farmers to have access to antimicrobials without a prescription for animal use contradicting the stringent access to antimicrobials employed by Act 101 of 1965 where a prescription had to be issued by a doctor to access antimicrobials in human health and a veterinarian for therapeutic purposes in sick animals. This gap can significantly contribute to increased AMR in animal health which can be transferred to humans through food-producing animals as their systems are interconnected. Subsequently, this challenge may reverse the strides achieved in the human health sector where there is a stringent medicines regulatory authority monitoring the quality, access, and use of medicines to curb AMR. The South African Veterinary Council (SAVC) has recognized the challenges of dual registration and addressing it by working towards having a single Act to control access and use of antimicrobials in the veterinary sector as part of the One Health initiatives in South Africa [39].

Findings also revealed that although Eswatini had a new Medicines and Related Substances Act 9 of 2016 to control access and use of antimicrobials in human health; however, there were still challenges related to the sale of antimicrobials over the counter in retail pharmacies because of the lack of enforcement of the legislation resulting in a negative impact on AMR containment. The challenges of weak regulations, allowing easy access to antibiotics and inappropriate use are not unique to Eswatini but are similar to findings of other studies performed in Ghana and many other sub-Saharan African countries where there is easy access to antibiotics whose indiscriminate use has the potential to increase AMR [16,40]. Naing et al. 2021 also confirm that there is easy access to antimicrobials in LMICs due to less restricted regulations resulting in a high consumption of antimicrobials more than HICs and increased incidences of AMR [41]. The WHO GAP advises countries to strengthen regulatory enforcement and eliminate substandard or falsified medicines across all sectors [10]. Ultimately, inconsistent and poorly enforced regulations in both human and animal health sectors can reverse progress and contribute to the rise in AMR. In South Africa, the human health sector is employing strict regulations to the access and use of antimicrobials whilst their efforts are compromised by gaps in the animal health sector. Therefore, improving enforcement of medicines regulations in all One Health sectors is crucial for the successful implementation and sustainability [42].

Political commitment and multi-sectoral leadership are critical to driving the AMR agenda, mobilizing and allocating resources appropriately, and enabling action [43]. Both the literature and implementation experiences have shown that countries with AMR leadership at a sufficiently senior level to wield decision-making authority have made greater progress and sustained the momentum over time [43]. Political leadership from senior government officials is essential for prioritizing AMR, allocating resources, and maintaining momentum. Where AMR is deprioritized, as noted in South Africa, implementation suffers. These findings are consistent with the literature indicating that governments often allocate fewer resources and attention to AMR compared to other health programs like HIV, TB, or malaria [38]. Political commitment is crucial for resource mobilization and sensitization of a program for its recognition. Therefore, awareness campaigns aimed at politicians and influential stakeholders are crucial for increasing ownership of NAPs on AMR as it easy for other competing health priorities to remove AMR on the agenda [16,41,44].

## 5. Conclusions

Although South Africa and Eswatini are neighboring countries with different economic statuses, they are facing similar challenges with regard to the progress of implementing their NAPs on AMR in a One Health approach. The findings of this study underscore several critical gaps in governance, accountability, financing, regulation, and multisectoral ownership that hinder effective implementation. To keep the AMR high on national agendas, both countries need to revive political commitment and improve awareness across all One Health sectors. Governments should prioritize the costing of NAP activities and advocate for dedicated financial and human resources from both domestic budgets and implementing partners. This is essential to ensure the sustainability and effectiveness of AMR interventions. In Eswatini, enforcement regulations must be developed to operationalize the Medicines and Related Substances Act, thereby strengthening control over antimicrobial access and use. In South Africa, regulatory contradictions between Act 101 of 1965 and Act 36 of 1947 must be resolved to prevent inappropriate antimicrobial distribution in the veterinary sector. Furthermore, both countries should increase representation of the environmental sector in national AMR coordinating committees. This would ensure ownership of environmental health responsibilities and better integration of AMR activities across sectors. Future studies are needed to assess whether there has been measurable progress in implementing NAPs on AMR in South Africa and Eswatini since the time this study was conducted.

## Figures and Tables

**Table 1:** Summary of study participants’ profile.

	Sector	No. of Key Informants	Institution Affiliation	Key Informants Per Institution	Total
	Health	9	National Department of Health	2	
			Academia	4	
			Research	2	
			National Health Laboratory Service	1	
**South Africa**	Agriculture	8	Department of Forestry, Fisheries and Environment	2	
			Veterinary Medicine Manufacturing Industry	1	
			South African Veterinary Services	1	
			Academia	1	
			Civic Society Organizations	2	
			World Organization for Animal Health (WOAH) representative	1	
	Environment	3	Academia	2	
			Water Research Commission	1	
**Eswatini**	Health	9	Ministry of Health	7	16
			WHO representative	1	
			National Health Laboratory Services	1	
	Agriculture	4	WOAH representative	1	
			Ministry of Agriculture	3	
	Environment	3	Ministry of Natural Resources (Department of Water Affairs)	3	

NB: Under the Department of Health and Ministry of Health, key informants included pharmacists, infectious disease specialists, microbiologists, medical laboratory specialists, and infection prevention specialists.

## Data Availability

The data presented in this study is available on request from the corresponding author. It cannot be made publicly available due to confidentiality and ethical reasons.

## Abbreviations

**AMR**	Antimicrobial resistance
**CARRO**	Coordination, Accountability, Resourcing, Regulation, Ownership
**DALRRD**	Department of Agriculture, Land Reform and Rural Development
**GAP**	Global Action Plan
**HIC**	High Income Countries
**IHR**	International Health Regulatory
**KI**	Key Informants
**LMICs**	Low Middle-Income Countries
**MAC**	Ministerial Advisory Committees
**MOH**	Ministry of Health
**NAMRCC**	National Antimicrobial Resistance Containment Committee
**NAP**	National Action Plan
**NDOH**	National Department of Health
**OH**	One Health
**QDA**	Qualitative Data Analysis
**SAVC**	South African Veterinary Council
**UHC**	Universal Health Coverage
**UMICs**	Upper Middle-Income Countries
**WHA**	World Health Assembly
**WHO**	World Health Organization
**WOAH**	World Organization for Animal Health

## References

[1] FAO, OIE, UNEP, WHO (2022). Antimicrobial Resistance Multi-Partner Trust Fund Terms of Reference: Combatting the Rising Global Threat of AMR Through a One Health Approach. https://mptf.undp.org/sites/default/files/documents/40000/mptf_amr_tor_april_2022_revision_2.1_final_incl_updated_toc.pdf..

[2] The Lancet (2024). Antimicrobial Resistance; an Enormous, Growing and Unevenly Distributed Threat to Global Health. https://www.thelancet.com/pb-assets/Lancet/infographics/antibiotic-resistance-2024/antibiotic-resistance2024-1720780667507.pdf.

[3] World Bank Group (2017). Drug-Resistant Infections. A Threat to Our Economic Future. https://documents1.worldbank.org/curated/en/323311493396993758/pdf/final-report.pdf.

[4] Lota M.M.M., Chua A.Q., Azupardo K. (2022). A Qualitative study on the design and implementation of the national action plan on antimicrobial resistance in the Philippines. Antibiotics.

[5] O’Neill J. (2016). Tackling Drug-Resistant Infections Globally: Final Report and Recommendations. https://amr-review.org/sites/default/files/160518_Final%20paper_with%20cover.pdf.

[6] World Health Organization (2024). All for Health, Health for All. WHO Investment Case Deep Dive: Antimicrobial Resistance. https://cdn.who.int/media/docs/default-source/documents/who-investment-case-amr-deep-dive.pdf?sfvrsn=ca1bd2e1_4&download=true.

[7] Word Health Organization (2019). Turning Plans into Action for Antimicrobial Resistance. https://iris.who.int/bitstream/handle/10665/311386/WHO-WSI-AMR-2019.2-eng.pdf?sequence=1.

[8] Nunes J.d.O., Domingues R.A.S., Barcellos R.M.P.C., Alves B.M.C.S., Carvalho I.P.S.F., de Tavares N.U.L. (2022). Policy and strategies addressing prevention and control of antimicrobial resistance in Brazil: A scoping review protocol. PLoS ONE.

[9] World Health Organization (2018). Monitoring Global Progress on Addressing Antimicrobial Resistance. Analysis Report of the Second Round of Results of AMR Country Self-Assessment Survey. https://www.who.int/publications/i/item/monitoring-global-progress-on-addressing-antimicrobial-resistance.

[10] World Health Organization (2015). Global Action Plan on Antimicrobial Resistance. https://www.who.int/publications/i/item/9789241509763.

[11] Munkholm L., Rubin O. (2020). The global governance of antimicrobial resistance: A cross-country study of alignment between the global action plan and national action plans. Glob. Health.

[12] World Health Organization Regional Office for Africa (2023). Regional Training and Workshop on the WHO Costing and Budgeting Tool for Antimicrobial Resistance National Action Plans. https://www.afro.who.int/publications/regional-training-and-workshop-who-costing-and-budgeting-tool-antimicrobial-resistance.

[13] Corrêa J.S., Zago L.F., Da Silva-Brandão R.R., de Oliveira S.M., Fracolli L.A., Padoveze M.C. (2023). The governance of antimicrobial resistance in Brazil: Challenges for developing and implementing a one health agenda. Glob. Public Health.

[14] World Health Organization (2022). Malawi National Action Plan on Antimicrobial Resistance. Review of Progress in the Human Health Sector. https://www.who.int/publications/i/item/9789240056848.

[15] Glover R.E., Naylor N.R. (2023). The WHO costing and budgeting tool for national action plans on antimicrobial resistance—A practical addition to the WHOle toolkit. JAC Antimicrob. Resist..

[16] Jimah T., Ogunseitan O. (2020). National action plan on antimicrobial resistance: Stakeholder analysis of implementation in Ghana. J. Glob. Health Rep..

[17] Shabangu K., Essack S.Y., Duma S.E. (2023). Barriers to implementing national action plans on antimicrobial resistance using a One Health approach: Policy makers’ perspectives from South Africa and Eswatini. J. Glob. Antimicrob. Resist..

[18] Adisasmito W.B., Almuhairi S., Behravesh C.B., Bilivogui P., Bukachi S.A. (2022). One Health: A new definition for a sustainable and healthy future. PLoS Pathog..

[19] Statistics South Africa (2022). Census 2022 Statistical Release P0301.4. https://census.statssa.gov.za/assets/documents/2022/P03014_Census_2022_Statistical_Release.pdf.

[20] UNICEF (2022). Eswatini Country Office Annual Report. https://data.unicef.org/country/swz.

[21] Department of Health, Department of Agriculture (2018). South African Antimicrobial Resistance National Strategy Framework; a One Health Approach 2018–2024. https://cdn.who.int/media/docs/default-source/antimicrobial-resistance/amr-spc-npm/nap-library/south-africa-antimicrobial-resistance-national-action-plan-2018---2024.pdf?sfvrsn=533118b0_1&download=true.

[22] Department of Health (2014). Antimicrobial Resistance National Strategy Framework 2014–2024. https://www.health.gov.za/wp-content/uploads/2024/11/Antimicrobial-Resistance-National-Strategy-Framework-2014-2024.pdf.

[23] WHO, Ukaid, The Fleming Fund, Mott McDonald, ICAP (2015). National Antimicrobial Resistance Containment Strategic Plan 2018–2022. https://www.who.int/publications/m/item/eswatini-national-antimicrobial-resistance-containment-strategic-plan-2018-2022.

[24] FAO, UNEP, WHO, WOAH (2023). Tracking AMR Country Self Assessment Survey (TrACSS) 2023 Country Report South Africa. https://amrcountryprogress.org/download/profiles/2023/english/EN_ZAF_TrACSS_2023_South-Africa.pdf.

[25] FAO, UNEP, WHO, WOAH (2023). Tracking AMR Country Self Assessment Survey (TrACSS) 2023 Country Report Eswatini. https://amrcountryprogress.org/download/profiles/2023/english/EN_SWZ_TrACSS_2023_Eswatini.pdf.

[26] World Medical Association (2014). Ethics governing medical negligence in clinical practice. J. Clin. Res. Bioeth..

[27] Mathews M., Huberman M. (1994). Qualitative Data Analysis.

[28] Creswell J.W. (2013). Qualitative Inquiry & Research Design: Choosing Among Five Approaches.

[29] Braun V., Clarke V. (2006). Using thematic analysis in psychology. Qual. Res. Psychol..

[30] Mgolozeli S.E., Duma S.E. (2020). “They destroyed my life because I do not feel like a man anymore”: An interpretative phenomenological analysis of Men’s lived experiences of rape victimization. Heliyon.

[31] World Health Organization (2022). WHO Implementation Handbook for National Action Plans on Antimicrobial Resistance. https://www.who.int/publications/i/item/9789240041981.

[32] Essack S. (2021). Water, sanitation and hygiene in national action plans for antimicrobial resistance. Bull. World Health Organ..

[33] Frumence G., Mboera L.E.G., Katale B.Z. (2021). Policy actors and human and animal health practitioners’ perceptions of antimicrobial use and resistance in Tanzania: A qualitative study. J. Glob. Antimicrob. Resist..

[34] Ogyu A., Chan O., Littmann J., Pang H.H., Lining X., Liu P. (2020). National action to combat AMR: A One-Health approach to assess policy priorities in action plans. BMJ Glob. Health.

[35] The Fleming Fund Terms of Reference for Request for Proposals. First Fleming Fund Country Grant to Eswatini 2020. https://www.flemingfund.org/countries/eswatini/.

[36] Frumence G., Mboera L.E.G., Sindato C., Katale B.Z., Kimera S. (2021). The governance and implementation of the national action plan on antimicrobial resistance in Tanzania: A qualitative study. Antibiotics.

[37] Gandra S., Alvarez-Uria G., Turner P., Joshi J. (2020). Antimicrobial resistance surveillance in Low- and Middle- Income countries: Progress and challenges in eight South Asian and Southeast Asian countries. Clin. Microbiol. Rev..

[38] Singh S.R., Chua A.Q., Tan S.T., Tam C.C. (2019). Combating antimicrobial resistance in Singapore: A qualitative study exploring the policy context, challenges, facilitators, and proposed strategies. Antibiotics.

[39] World Health Organization (2018). Tackling Antimicrobial Resistance Together. Working Paper 1.0: Multisectoral Coordination. https://www.who.int/publications/i/item/tackling-antimicrobial-resistance-together-working-paper-1.0-multisectoral-coordination.

[40] Koduah A., Gyansa-Lutterodt M., Hedidor G.K., Sekyi-Brown R., Asiedu-Danso M. (2021). Antimicrobial resistance national level dialogue and action in Ghana: Setting and sustaining the agenda and outcomes. One Health Outlook.

[41] Schellack N., Brink A., Mendelson M. (2017). A Situational analysis of current antimicrobial governance, regulation, and utilization in South Africa. Int. J. Infect. Dis..

[42] Mokwele R.N., Schellack N., Bronkhorst E. (2022). Using mystery shoppers to determine practices pertaining to antibiotic dispensing without a prescription among community pharmacies in South Africa—A pilot survey. JAC Antimicrob. Resist..

[43] Naing S., van Wijk M., Vila J., Ballesté-Delpierre C. (2021). Understanding antimicrobial resistance from the perspective of public policy: A multinational knowledge, attitude, and perception survey to determine global awareness. Antibiotics.

[44] Thomas S.A., Mathew P., Ranjalkar J., Nguyen T.B., Van Giao V.T.Q., Chandy S.J. (2024). Public perception and community-level impact of national action plans on antimicrobial resistance in Vietnam. JAC Antimicrob. Resist..

